# Respiratory muscle dysfunction in long-COVID patients

**DOI:** 10.1007/s15010-022-01840-9

**Published:** 2022-05-16

**Authors:** Jan K. Hennigs, Marie Huwe, Annette Hennigs, Tim Oqueka, Marcel Simon, Lars Harbaum, Jakob Körbelin, Stefan Schmiedel, Julian Schulze zur Wiesch, Marylyn M. Addo, Stefan Kluge, Hans Klose

**Affiliations:** 1 Division of Pneumology, II. Department of Medicine, University Medical Center Hamburg-Eppendorf, Martinistrasse 52, 20246 Hamburg, Germany; 2 Division of Infectious Diseases, I. Department of Medicine, University Medical Center Hamburg-Eppendorf, 20246 Hamburg, Germany; 3 Department of Intensive Care Medicine, University Medical Center Hamburg-Eppendorf, 20246 Hamburg, Germany; 4 Department of Clinical Immunology of Infectious Diseases, Bernhard Nocht Institute for Tropical Medicine, 20359 Hamburg, Germany; 5 German Center for Infection Research (DZIF), Partner Site Hamburg-Lübeck-Borstel-Riems, 20359 Hamburg, Germany

**Keywords:** SARS-CoV-2, COVID-19, *P*0.1, *P*Imax, *P*0.1/*P*Imax, Long COVID

## Abstract

**Purpose:**

Symptoms often persistent for more than 4 weeks after COVID-19—now commonly referred to as ‘Long COVID’. Independent of initial disease severity or pathological pulmonary functions tests, fatigue, exertional intolerance and dyspnea are among the most common COVID-19 sequelae. We hypothesized that respiratory muscle dysfunction might be prevalent in persistently symptomatic patients after COVID-19 with self-reported exercise intolerance.

**Methods:**

In a small cross-sectional pilot study (*n* = 67) of mild-to-moderate (nonhospitalized) and moderate-to-critical convalescent (formerly hospitalized) patients presenting to our outpatient clinic approx. 5 months after acute infection, we measured neuroventilatory activity *P*_0.1_, inspiratory muscle strength (*P*I_max_) and total respiratory muscle strain (*P*_0.1_/*P*I_max_) in addition to standard pulmonary functions tests, capillary blood gas analysis, 6 min walking tests and functional questionnaires.

**Results:**

Pathological *P*_0.1_/*P*I_max_ was found in 88% of symptomatic patients. Mean *P*I_max_ was reduced in hospitalized patients, but reduced *P*I_max_ was also found in 65% of nonhospitalized patients. Mean *P*_0.1_ was pathologically increased in both groups. Increased *P*_0.1_ was associated with exercise-induced deoxygenation, impaired exercise tolerance, decreased activity and productivity and worse Post-COVID-19 functional status scale. Pathological changes in *P*_0.1_, *P*I_max_ or *P*_0.1_/*P*I_max_ were not associated with pre-existing conditions.

**Conclusions:**

Our findings point towards respiratory muscle dysfunction as a novel aspect of COVID-19 sequelae. Thus, we strongly advocate for systematic respiratory muscle testing during the diagnostic workup of persistently symptomatic, convalescent COVID-19 patients.

## Introduction

While lung, kidney and the vascular system appear to be the main sites of acute Severe acute respiratory syndrome coronavirus type 2 (SARS-CoV-2)-related complications [1, 2], early sequelae of coronavirus disease (COVID)-19 are reported by the vast majority of convalescent patients [3, 4]. Sequelae persisting for longer than 4 weeks are now phenotypically summarized under the umbrella term “Long COVID” [4]. The most commonly reported symptoms include persistent dyspnea and fatigue in up to 51% and 63% of cases, respectively, which are also among the longest lasting sequelae [3, 5]. As recently reported, exertional intolerance and dyspnea can also be observed in Long COVID patients with preserved lung function [6]. In this light, in addition to a growing body of evidence regarding pulmonary parenchymal and cardiac sequelae, exercise intolerance in Long COVID patients might have additional causes related to respiratory muscular dysfunction.

In a cross-sectional approach, we have therefore prospectively investigated respiratory drive and effort in Long COVID-19 patients with self-reported exercise intolerance presenting to our outpatient department (OPD).

## Methods

Sixty-seven adult convalescent COVID-19 patients (30 female, 37 male, mean age: 49 years, baseline characteristics are given in Table 1) presenting after mild to critical disease (according to World Health Organization (WHO) classification) completed general symptom, activity and productivity (modified Work Productivity and Activity Impairment (WPAI) score) questionnaires before undergoing complete pulmonary function testing (PFT), including spirometry, body plethysmography, capillary blood gas analyses (CBG) at rest and immediately after performing a 6 min walk test (6MWT). Assessment of dyspnea intensity at rest and during the 6MWT using the Modified BORG Dyspnea Scale (Borg CR10) was performed. In addition, respiratory muscle testing to assess respiratory drive and effort was conducted following current guidelines [7, 8]. All adult patients with persisting symptoms for ≥ 4 weeks after COVID-19 with a proven record of SARS-CoV-2 infection (positive PCR for SARS-CoV-2 or presence of SARS-CoV-2-specific nucleocapsid antibodies) were eligible after informed consent. Patients < 18 years or without proven SARS-CoV-2 infection were excluded. Patients were recruited via the Post COVID Clinics of the Divisions of Pneumology and Infectious Diseases at the University Medical Center Hamburg Eppendorf. Eligible patients categorized as hospitalized had to be hospitalized due to COVID-19.

**Table 1:** Baseline characteristics of the study cohort at the time of presentation to the outpatient department

Hospitalization during COVID-19	No *n* = 30	Yes *n* = 37	*p* value
Age (years) Mean ± SD	41.1 ± 10.7	55.9 ± 12.5	< 0.001
Sex (*n*, %)			
Female	17 (56.7)	13 (35.1)	0.130
BMI (kg/m^2^) mean ± SD	25.3 ± 4.5	28.6 ± 5.3	< 0.001
Time from Dx (days) mean ± SD	123.6 ± 69.4	147.5 ± 70.8	0.170
Smoking status (*n*, %)			0.322
Active	4 (13.3)	2 (5.4)	
Former	8 (26.7)	16 (43.2)	
Never	18 (60.0)	18 (48.6)	
Unknown	0 (0.0)	1 (2.7)	
Disease severity (*n*, %)			< 0.001
WHO class			
Mild	20 (66.7)	2 (5.4)	
Moderate	10 (33.3)	12 (32.4)	
Severe	0 (0.0)	7 (18.9)	
Critical	0 (0.0)	16 (43.2)	
ARDS (*n*, %)			
yes	0 (0.0)	15 (40.5)	< 0.001
Total no. of comorbidities median (IQR)	0 (0–1)	2 (0–3)	< 0.001
Comorbidities (*n*, %)			
Diabetes	0 (0)	6 (16.7)	0.028
Cardiovascular disease	2 (6.7)	9 (24.3)	0.043
Hypertension	4 (13.3)	13 (35.1)	0.037
Renal insufficiency	0 (0)	5 (13.9)	0.011
Adipositas	0 (0–0)	0 (0–1)	0.030
Liver disease	0 (0–0)	0 (0–1)	0.003
Thyroid dysfunction	2 (6.7)	4 (10.8)	0.550
Neurological disease / myopathies	0 (0)	0 (0)	–
Asthma	8 (26.7)	5 (13.5)	0.176
COPD	0 (0)	1 (2.7)	0.364
Other lung disease	1 (3.3)	1 (2.7)	0.880
PFT (%)			
Mean ± SD			
FVC	98.2 ± 12.4	83.7 ± 21.5	0.002
FEV1	97.2 ± 11.9	87.3 ± 18.0	0.012
FEV1/FVC	99.3 ± 8.0	105.7 ± 8.6	0.003
RV	107.4 ± 28.3	91.4 ± 28.4	0.025
TLC	103.5 ± 14.5	87.5 ± 18.9	< 0.001
FRC	96.3 ± 21.6	82.2 ± 21.9	0.012
DLCO	83.0 ± 12.6	68.8 ± 17.7	0.001
PFT Pattern (*n*, %)			
Restrictive	1 (3.3)	13 (35.1)	< 0.001
Obstructive	3 (10.0%)	1 (2.7%)	0.210
6MWT			
Mean ± SD			
6MWD (m)	607.0 ± 53.7	514.7 ± 127.2	< 0.001
CBG (mmHg)			
Median (IQR)			
Δ*P*_a_O_2_	1.5 (− 7.8–5.2)	− 7.8 (− 12.1–− 0.4)	0.021
Δ*P*_a_CO_2_	0.8 (− 0.8–2.4)	− 0.5 (− 1.1–− 2.2)	0.406
Dyspnea (Borg CR10)			
Median (IQR)			
Difference	1.00 (0.62–3.00)	2.00 (0.50–2.25)	0.984
Mean ± SD			
At rest	0.4 ± 0.8	0.6 ± 1.1	0.462
Exercise	2.2 ± 1.7	2.3 ± 1.7	0.829
Productivity (modified WPAI)			
Median (IQR)	5.5 (3.0–11.5)	10.0 (4.0–15.25)	0.104
PCFS Scale			
Median (IQR)	2 (1–3)	2 (1–3)	0.698

*SD* Standard deviation, *BMI* Body Mass Index, *Dx* Diagnosis, *IQR* Interquartile Range, *ARDS* Acute respiratory distress syndrome, *No* Number, *COPD*: chronic obstructive pulmonary disease, *PFT* Pulmonary Function Test, *FVC* Forced vital capacity, *FEV1* Forced expiratory volume in 1 s, *FEV1/FVC* Tiffeneau-Pinelli index, *RV* Residual volume, *TLC* Total lung capacity, *FRC* Functional residual capacity, *DLCO* Diffusing capacity for carbon monoxide, *6MWT* 6-min walk test, *CBG* Capillary blood gas, *Δ* Difference between Rest and Exercise, *CR* Category ratio, *WPAI* Work Productivity and Activity Index, *PCFS* Post-COVID-19 Functional Status

PRISM 9 (GraphPad Inc, San Diego, CA) and R for macOS version 4.0.3 (https://cran.r-project.org) with RStudio 1.3 (RStudio, Boston, MA) were used for the following statistical analyses: one-sample *t* test and Pearson correlation analysis for normally distributed data (D’Agostino-Pearson Test); Mann–Whitney, Spearman correlation and Fisher’s exact test for nonparametric data; corrplot 0.84 library was utilized for principal component clustering.

## Results

At the time of presentation to our OPD (median of 152 days, IQR: 65–260 after onset of acute symptoms), patients initially hospitalized due to COVID-19 (55% of cohort) showed reduced PFT parameters compared with nonhospitalized patients. In addition, initially hospitalized patients walked 92.3 m (15.2%) less in the 6MWT and showed a more pronounced decrease in the arterial partial pressure of oxygen (*P*_a_O_2_) during the 6MWT (median: + 1.5 mmHg vs. − 7.8 mmHg). No differences were found in dyspnea perception, functional impairment, daily activity or productivity. While hospitalized patients were older, had a higher body mass index and more comorbidities which were associated with more severe acute disease, history of lung disease was rare and did not differ between hospitalized and nonhospitalized patients (Table 1).

**Fig. 1:**
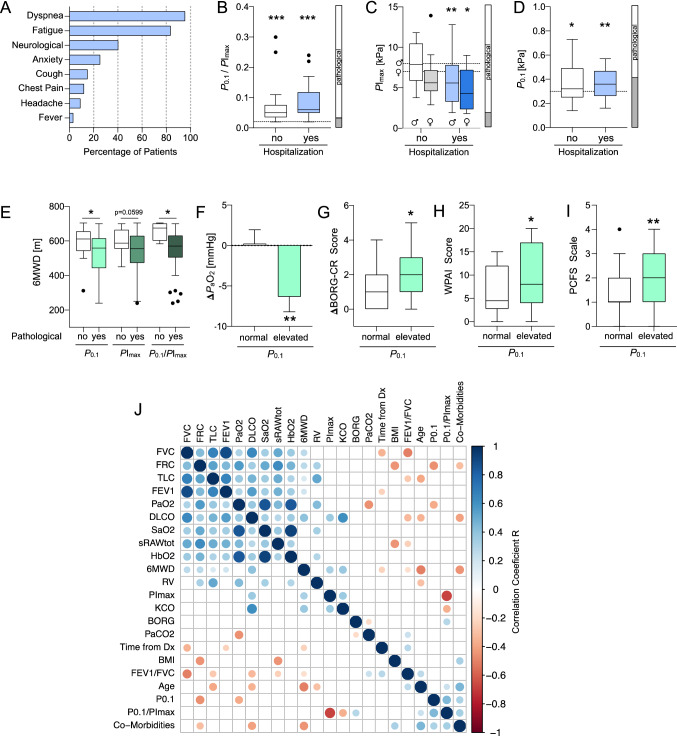
Respiratory muscle impairment after COVID-19 is associated with impaired exercise tolerance, exercise-induced deoxygenation, activity and functional outcome **A** Persisting symptoms of convalescent COVID-19 patients at the time of presentation to the outpatient department (OPD) (mean: 152 days after diagnosis, Dx, *n* = 67). **B** Respiratory muscle strain *P*_0.1_/*P*I_max_ at OPD presentation after COVID-19 by hospitalization status of acute COVID-19 (***p = 6.0E−08 and ***p = 5.8E−11, respectively; one-sample Wilcoxon test versus upper limit of normal cutoff: 0.02). **C** Inspiratory muscle strength *P*I_max_ by sex and hospitalization status (nonhospitalized: male (♂), *p* = 0.83 and female (♀), *p* = 0.10; hospitalized: male, **p = 0.0079; female, *p = 0.0269; one-sample Wilcoxon versus cutoff: 8 kPa, male and 7 kPa, female). Fractions of sex- and age-corrected pathological test results are given in the adjacent vertical bar. **D** Airway occlusion pressure at 0.1 s, *P*_0.1_ per same patient as in (B) (*p = 0.0291, **p = 0.0027, one-sample *t* test versus cutoff: 0.3 kPa) and fraction of pathological test results (adjacent bar). **E** Six-minute walking test (6MWT) distance (6MWD) in meters (m) by *P*_0.1_ (*p = 0.0219), *P*I_max_ (*p* = 0.0599) and *P*_0.1_/*P*I_max_ (*p* = 0.0162), Mann–Whitney test. **F** Difference in arterial partial pressures for oxygen (Δ*P*_a_O_2_) by *P*_0.1_ (**p = 0.0134, unpaired, 2-sided *t* test) **G** Difference in self-reported dyspnea perception (BORG-CR score) at rest and immediately after 6MWT by *P*_0.1_ (ΔBORG-CR, *p = 0.0299, Mann–Whitney test). **H** Self-reported activity and productivity impairment (modified WPAI score) in the last seven days before presentation to the OPD by *P*_0.1_ (*p = 0.0471, Mann–Whitney test). **I** Self-reported Post-COVID-19 Functional Status (PCFS) scale at the time of presentation to the OPD by *P*_0.1_ (**p = 0.0058, Mann–Whitney test). **J** Multivariate matrix of significantly (*p* < 0.05) correlated variables from the study cohort (Pearson or Spearman *R* values) sorted by first principal component. Box-and-whiskers showing medians + interquartal range (IQR) and outliers (Tukey method). In **F**, normally distributed data are given as mean ± standard error of the mean. Dashed lines in **G**, **H** and **I** represent pathological (sex-specific) cutoff values. Mann–Whitney test in **F**, **G**, **H** and **I** was used for comparison of groups with normal vs. elevated *P*_0.1_.Vertical bars in **B**, **C** and **D** represent the fraction of pathological (open) and normal (gray) values from the total cohort

In addition to exercise intolerance reported by all patients, the most frequent symptoms were persistent exertional dyspnea (95.5%) and fatigue (83.6%, Fig. 1A). These symptoms were associated with alterations in respiratory drive and effort. Both hospitalized and nonhospitalized patients had increased total respiratory muscle strain (= occlusion pressure at 0.1 s (*P*_0.1_)/ maximal inspiratory pressure (*P*I_max_) > 0.02); 97.2 vs. 87.1%, *P*_0.1_/*P*I_max_ range: 3–25%, *p* = 0.0005 and *p* = 6.6E-08, Fig. 1B) at the time of presentation to the OPD. Hospitalized patients showed a trend towards more pronounced respiratory muscle strain (*P*_0.1_/*P*I_max_: 0.05 vs. 0.06, *p* = 0.056). Inspiratory muscle strength (as determined by the peak value of maximum inspiratory mouth pressure measured from residual volume (*P*Imax_peak_ RV) = *P*I_max_) was decreased below six and age-specific cutoffs in 88% of patients (Fig. 1C, vertical bar), predominantly in patients previously hospitalized due to COVID-19 (*p* = 0.0108, female and *p* = 0.0079, male; Fig. 1C). In addition, inspiratory muscle weakness was more frequent in women (96.4 vs. 79.3%, *p* = 0.0088, Fisher’s exact test). Neuroventilatory activity as determined by *P*_0.1_ > 0.3 kPa (~ 3.1 cmH_2_O) was elevated in 56% of patients (mean *P*_0.1_: 0.36 and 0.37 kPa, *p* = 0.0291 and *p* = 0.0029, nonhospitalized and hospitalized, respectively, Fig. 1D), which was independent of hospitalization status (*p* = 0.64).

Clinically, alterations in respiratory drive and effort after COVID-19 were associated with reduced distance (6MWD) in the 6MWT (*P*_0.1_: 595.5 vs. 529.3 m, *p* = 0.0219; *P*I_max_: 600.1 vs. 537.6 m, *p* = 0.0599; *P*_0.1_/*P*I_max_: 659.3 vs. 548.5 m, *p* = 0.0162; Fig. 1E).

While no patient was hypoxemic at rest, convalescent COVID-19 patients with elevated *P*_0.1_ showed a significant decrease in arterial oxygen partial pressure (*P*_a_O_2_) during the 6MWT (Δ*P*_a_O_2_: − 6.6 mmHg, *p* = 0.0134; Fig. 1F). In all patients with exertional deoxygenation, pulmonary thromboembolic disease was ruled out by subsequent ventilation/perfusion scans.

Patients with elevated *P*_0.1_ after COVID-19 reported increased dyspnea during the 6MWT, as informed by a larger difference (Δ) in BORG scores at rest and upon exercise (+ 1.3 vs. + 2.1, *p* = 0.0299; larger = worse, Fig. 1G). In addition, patients with elevated *P*_0.1_ > 0.3 kPa also reported less daily activity and productivity due to persisting symptoms (modified WPAI score, 6.3 vs. 9.8, *p* = 0.0471; higher = larger impairment, Fig. 1H) as well as increased overall functional impairment as determined by the Post-COVID functional status (PCFS, [9]) scale (1 vs. 2, *p* = 0.0058; higher = larger impairment, Fig. 1I).

In univariate regression analysis, *P*_0.1_ was associated with functional residual capacity (FRC, *r* = − 0.27, *p* = 0.046), Δ*P*_a_O_2_ (*r* = − 0.30, *p* = 0.007), number of comorbidities (*r* = 0.27, *p* = 0.044) and *P*_0.1_/*P*I_max_ (*r* = 0.30, *p* = 0.007). *P*I_max_ was correlated with the diffusing capacity of carbon monoxide (DLCO, *r* = 0.37, *p* = 0.006), 6MWD (*r* = 0.33, *p* = 0.014), Carbon monoxide transfer coefficient (KCO, *r* = 0.36, *p* = 0.006) and *P*_0.1_/*P*I_max_ (*r* = − 0.54, *p* = 2.1E-05). *P*_0.1_/*P*I_max_ was associated with KCO (*r* = − 0.33, *p* = 0.015), ΔBORG score (*r* = 0.33, *p* = 0.013), age (*r* = 0.26, *p* = 0.05) and number of comorbidities (*r* = 0.40, *p* = 0.003) (Fig. 1J).

In a principal component-based multivariate analysis, *P*_0.1_ and *P*_0.1_/*P*I_max_ clustered with age, body-mass-index (BMI), number of comorbidities, FEV1/FVC, time from diagnosis and CBG while *P*I_max_, did not clearly cluster with any of the parameters (Fig. 1J).

Comorbidities were not associated with pathologically altered *P*_0.1_, *P*I_max_ or *P*_0.1_/*P*I_max_ (all *p* > 0.05). Patients with a history of asthma were less likely to show pathological *P*_0.1_/*P*I_max_ (*χ*^2^ = 5.41, *p* = 0.020).

## Discussion

In our cross-sectional pilot study of convalescent COVID-19 patients with persistent exercise intolerance, we identified a high prevalence of impaired respiratory muscle function and upregulated neuroventilatory activity ~ 5 months after diagnosis. Functionally, this was associated with reduced 6MWD and daily activity/productivity in connection with exercise-induced deoxygenation.

Recently, published PFT data of COVID-19 patients show reduced TLC and DLCO up to 6 months after infection, which occurred more often in patients with severe disease [5]. This is in line with our data showing that patients initially hospitalized for COVID-19 had significantly lower PFT parameters, including TLC and DLCO, up to 5 months after infection. This was also associated with reduced exercise capacity in hospitalized patients after COVID-19 as measured by 6MWD. Our study extends these findings, as we report a high prevalence of increased respiratory drive and impaired respiratory muscle capacity in convalescent, persistently symptomatic COVID-19 patients.

In our cohort, patients requiring hospitalizations, including ICU treatment, also had impaired respiratory muscle strength as demonstrated by reduced *P*I_max_, which is consistent with recently reported findings of fibrotic diaphragm remodeling in patients who died due to COVID-19-related ARDS [10].

Elevated *P*_0.1_, as found in the majority of our patients, is strongly associated with heightened dyspnea perception [11]. This was also the case in our cohort, as shown by elevated BORG-CR scores and everyday activity, productivity and COVID-related functional impairment (PCFS). Strikingly, this was not only the case in hospitalized patients where elevated *P*_0.1_ might be a consequence of reduced inspiratory muscle strength *P*I_max_ but also in nonhospitalized patients.

Therefore, our data support that, pathophysiologically, elevated *P*_0.1_ might be a function of exercise-induced deoxygenation in convalescent, persistently symptomatic COVID-19 patients. While pulmonary thromboembolic disease was not detected by V/Q scan (as described above), six patients showed signs of ground-glass opacity and (mostly minor) fibrotic changes and exercise-induced deoxygenation was associated with lower DLCO (Fig. 1J). Systematic analysis of these changes, however, was out of the scope of the present study, which is a limitation. Additionally, due to unavailability of data in some patients, we cannot exclude pre-existing changes in respiratory drive and effort sustained from before SARS-CoV-2 infection. Additional limitations include putatively biased patient selection, as most patients reported to our OPD with persistent symptoms after COVID-19, with very few patients referred for routine follow-up after COVID-19. Patients and staff were also not blinded to the overall testing, possibly inserting additional bias in the measurement as does lack of historical PFT data. Particularly for ICU patients, muscular deconditioning associated with intensive care might contribute to respiratory muscle impairment. Although it was not possible to differentiate inspiratory muscle impairment from generalized muscle weakness or postinfection myopathy, in our cohort, creatine kinase and myoglobin serum levels did not differ between patients with normal or abnormal respiratory muscle function (*p* = 0.202 and *p* = 0.075, respectively). In addition, pre-existing conditions/comorbidities did not correlate with abnormal respiratory muscle function in our cohort. Also, inspiratory muscle weakness also occurred frequently in nonhospitalized patients (65%). We also cannot specifically attribute the detected changes in respiratory drive and inspiratory muscle function to SARS-CoV-2, as we cannot rule out a general effect of viral infections. Regardless of SARS-CoV-2 specificity, the high prevalence in our pilot study points toward a relevant healthcare burden given the pandemic nature of COVID-19.

As there is strong evidence that chronic fatigue syndrome (CFS) is associated with COVID-19 [3, 5], it is compelling to speculate to what extent heightened neuroventilatory activity, as documented by *P*_0.1_ in our cohort, contributes to COVID-19-CFS. Particularly, the inability to adequately increase respiratory effort upon increased respiratory drive is known to worsen respiratory distress [11]. Therefore, more invasive techniques such as twitch interpolation might help to further characterize dysregulation of respiratory drive and effort in Long COVID patients.

## Conclusion

We were able to detect increased respiratory drive as well as inspiratory muscle dysfunction in persistently symptomatic patients approx. 5 months after COVID-19. Notwithstanding the small sample size, our findings reveal a previously unidentified neuromuscular component of COVID-19 sequelae.

Given the wide accessibility of respiratory muscle testing as a relatively low-cost approach (in particular in comparison with imaging and immunological laboratory studies), we strongly advocate for systematic respiratory muscle testing in the diagnostic workup of persistently symptomatic, convalescent COVID-19 (Long COVID) patients.

## Data Availability

Data analyzed during this study are available from the corresponding authors upon reasonable request.

## Abbreviations

ARDS: Acute respiratory distress syndrome
BMI: Body-Mass-Index
CBG: Capillary blood gas
Δ: Difference between rest and exercise
CR: Category ratio
Dx: Diagnosis
DLCO: Diffusion capacity for carbon monoxide
FEV1: Forced expiratory volume in 1 s
FRC: Functional residual capacity
FVC: Forced vital capacity
IQR: Inter-Quartile Range
*P*_0.1_: Airway occlusion pressure at 100 ms
*P*I_max_: Maximum inspiratory mouth pressure
*P*I_maxpeak_ RV: Peak value of maximum inspiratory mouth pressure measured from residual volume
PCFS: Post-COVID-19 functional status
PFT: Pulmonary Function Test
RV: Residual volume
6MWT: 6-Minute walk test
6MWD: 6-Minute walking distance
TLC: Total lung capacity
WPAI: Work Productivity and Activity Index
